# Impact of Hypothyroidism on Echocardiographic Characteristics of Patients With Heart Valve Disease: A Single-Center Propensity Score-Based Study

**DOI:** 10.3389/fendo.2020.554762

**Published:** 2020-09-24

**Authors:** Tianyu Zhai, Zhenqin Cai, Jiayu Zheng, Yan Ling

**Affiliations:** ^1^Department of Endocrinology and Metabolism, Zhongshan Hospital, Fudan University, Shanghai, China; ^2^Department of Cardiovascular Surgery, Zhongshan Hospital, Fudan University, Shanghai, China

**Keywords:** hypothyroidism, heart valve disease (HVD), propensity score (PS), echocardiographic characteristics, left atrial enlargement

## Abstract

**Background:** Hypothyroidism is known to be correlated with multiple heart diseases. However, the influence of hypothyroidism on the patients with heart valve disease (HVD) is still unclear. The purpose of our study was to investigate the impact of hypothyroidism on echocardiographic characteristics of patients with heart valve disease.

**Methods:** We conducted a retrospective cohort study which included 2,128 patients with HVD, and they were divided into euthyroid, subclinical hypothyroidism (SCHypoT), and overt hypothyroidism (OHypoT) group. Echocardiographic characteristics before and after valve surgery between groups were compared by using propensity score (PS) analysis. Kaplan–Meier analysis was used to compare the percent of recovery of left atrial (LA) enlargement between groups.

**Results:** Overall, 463 patients had hypothyroidism (404 SCHypoT patients and 59 OHypoT patients), and 1,665 patients were euthyroid. At baseline, hypothyroidism was associated with significantly higher left atrial diameter (LAD), interventricular septum thickness, left ventricular posterior wall thickness, pulmonary artery systolic pressure, and lower left ventricular ejection fraction. After valve surgery, only LAD remained significantly higher in the patients with hypothyroidism. Additionally, patients with hypothyroidism had a significantly lower recovery rate of LA enlargement after valve surgery compared with euthyroid patients.

**Conclusion:** Hypothyroidism was associated with a larger LAD in patients with HVD before and after surgery, which may suggest that hypothyroidism is a risk factor of LA enlargement of HVD. Besides, hypothyroidism was associated with a significantly lower recovery rate of LA enlargement after valve surgery.

## Introduction

The cardiovascular system or myocardium is an important target of thyroid hormones, which exert many regulatory effects on heart rate, cardiac output, blood volume, vascular resistance, and tissue oxygen consumption (1). Hypothyroidism is relatively common in thyroid disorders, and some previous studies have illustrated that hypothyroidism correlates with multiple heart diseases, such as congestive heart failure, coronary atherosclerosis, and myocardial infarction (2–4). Moreover, a higher risk of cardiac mortality was found to be associated with hypothyroidism in the general population (5).

In the past few decades, the impact of thyroid dysfunction on the cardiovascular system has received widespread attention. Evidence from several studies showed that subclinical and overt hypothyroidism significantly affected the electrocardiographic variables and atrial or ventricular function. The patients with hypothyroidism were found to display higher QT dispersion, lower heart rate variability (HRV), and aberrant diastolic velocities (6–8); levothyroxine treatment for hypothyroidism may bring cardiac benefits (6, 9). Additionally, the adverse effect of hypothyroidism on cardiac structure has been demonstrated by some researchers, such as larger interventricular septum (IVS) thickness, left ventricular posterior wall (LVPW) thickness, and decreased left ventricular ejection fraction (LVEF), and they also confirmed the significant improvement after levothyroxine treatment (10–20). A retrospective cohort study found that in patients with dilated cardiomyopathy (DCM), patients with subclinical hypothyroidism had larger left ventricular (LV) and left atrial (LA) diameter and higher all-cause mortality (21). However, there are also inconsistent conclusions. In the original cohort of the Framingham Heart Study, no significant associations were identified between TSH concentrations and LA diameter or LV structure in 1,376 participants.

Heart valve disease (HVD) is characterized as valvular stenosis or regurgitation, which may induce important changes including ventricle or atrium enlargement, myocardial hypertrophy, and even cardiac failure. Several risk factors, including infectious, inflammatory, autoimmune, genetic, and oxidative stress, are associated with the development of HVD (22–24). However, the influence of hypothyroidism on the cardiac structure of patients with HVD remains unclear. Here, we conducted a single-center, retrospective cohort study which includes 2,128 HVD patients and applied propensity score analyses to investigate the impact of hypothyroidism on echocardiographic characteristics at the baseline. After valve surgery, follow-up information of 1,327 HVD patients was acquired and further analyzed. The large sample size and the in-depth statistical analysis of the data provided greater reliability of our study.

## Materials and Methods

### Study Populations

**Figure 1:**
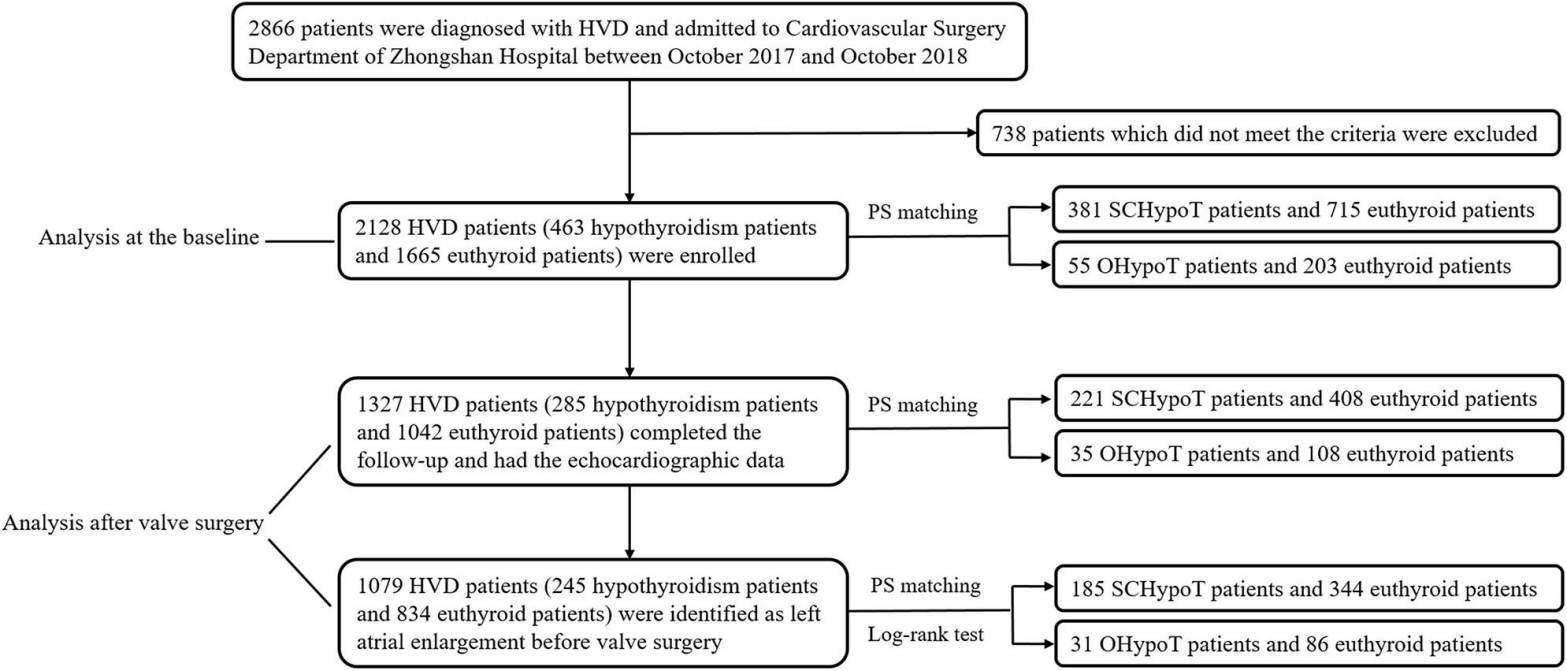
Flowchart of study subject recruitment and analysis. HVD, heart valve disease; PS, propensity score; SCHypoT, subclinical hypothyroidism; OHypoT, overt hypothyroidism.

Between October 2017 and October 2018, we enrolled consecutive patients who were diagnosed with HVD and admitted to Cardiovascular Surgery Department of Zhongshan Hospital, which is affiliated to Fudan University in Shanghai. The clinical data of all 2,866 patients were obtained through medical record review. We excluded patients according to the following criteria: (1) patients who had undergone valve surgery before the enrollment, (2) patients who were diagnosed with hyperthyroidism, (3) patients who did not have thyroid function measurement, (4) patients who used medications (antithyroid drugs, thyroid hormone, amiodarone, and glucocorticoid hormone) influencing thyroid function, and (5) patients with missing data. Finally, this study included 2,128 (1,152 males, 976 females) patients for analysis (Figure 1).

The study protocol was approved by the ethics committee of Zhongshan Hospital of Fudan University, and informed consents were obtained from all patients.

### Clinical Information Collection and Laboratory Measurements

Clinical information about medical history and health-related behaviors of all patients was recorded and checked by two physicians. The clinical variables included age, sex, weight, height, body mass index (BMI), systolic/diastolic blood pressure, New York Heart Association (NYHA) functional class, comorbidities, smoking, drinking, percutaneous coronary intervention (PCI), medications, surgery, and hospital/intensive care unit (ICU) length of stay. Comorbidities included hypertension, diabetes mellitus, congestive heart failure, coronary artery disease, atrial flutter/fibrillation, cerebrovascular disease, chronic kidney disease, and chronic liver disease. Medications included angiotensin-converting enzyme inhibitors (ACEIs)/angiotensin II receptor blockers (ARBs) and statins. Diagnoses of HVD were made by transthoracic or transesophageal echocardiography according to the guidelines (25, 26).

Serum-free T_3_ (fT_3_), free T_4_ (fT_4_), and TSH measurement were performed by a Modular E170 automatic electrochemiluminescence analyzer (Roche Diagnostics Ltd., Germany) using the electrochemical luminescence method. The normal range for fT_3_, fT_4_, and TSH is 2.8–7.1 pmol/L, 12–22 pmol/L, and 0.27–4.20 mIU/L, respectively.

Two-dimensional-guided M-mode and Doppler echocardiography was performed by iE33 Echocardiography (Philips Medical Systems, Bothell, WA, USA). Echocardiographic measurements of aortic root diameter (ARD), left atrial diameter (LAD), left ventricular end-diastole diameter (LVEDD), and end-systole diameter (LVESD); interventricular septum (IVS) thickness; left ventricular posterior wall (LVPW) thickness; and pulmonary artery systolic pressure (PASP) were obtained. The left ventricular ejection fraction (LVEF) was calculated as follows: LVEF (%) = (LVEDD-LVESD)/LVEDD × 100%. The normal range for echocardiographic measurements was listed as the following: ARD (20–37 mm), LAD (19–40 mm), LVEDD (35–56 mm), LVESD (23–35 mm), IVS thickness (6–11 mm), LVPW thickness (6–11 mm), PASP (<40 mmHg), and LVEF (55–80%).

### Follow-Up

Follow-up was performed in the patients undergoing valve surgery. Information was collected from a retrospective review of the medical records. Follow-up was completed in 62.4% (1,327/2,005) patients with a median follow-up period of 5.0 (range: 3–18 months) months.

### Propensity Score Analysis

According to the thyroid hormone levels, all patients were divided into the euthyroid group (normal TSH, FT_3_, and FT_4_) and hypothyroidism group (TSH > 4.2 mIU/L), which was further divided into the subclinical hypothyroidism (SCHypoT) group (4.2 < TSH <10.0 mIU/L) and overt hypothyroidism (OHypoT) group (TSH ≥ 10.0 mIU/L). We adopted propensity score (PS) matching to minimize the imbalance in the potential confounders between euthyroid and hypothyroidism groups. PS was calculated by multivariable logistic regression models, which assessed the propensity for developing hypothyroidism. In the matching of baseline characteristics, the logistic regression model included the covariates of age, sex, weight, height, smoking, drinking, percutaneous coronary intervention (PCI), medications, hypertension, diabetes mellitus, congestive heart failure, coronary artery disease, atrial flutter/fibrillation, cerebrovascular disease, chronic kidney disease, and chronic liver disease. NYHA class before surgery, valvular surgery type, and follow-up period were added into the model for follow-up matching. To investigate the effects of severity of hypothyroidism on HVD, we further performed 1:2 and 1:4 nearest neighbor matching for SCHypoT and OHypoT groups, respectively. A caliper was chosen as 0.02 because it ensures the reasonable balance of covariates and does not lose many unmatchable patients. Standardized differences (SD) were used to assess the balance of covariates in PS matching.

### Statistical Analyses

Continuous variables were reported as mean ± standard deviation or median (interquartile range), and categorical variables were represented by frequency and percentage. Differences between normally distributed continuous variables were assessed using the Student's *t*-test. The Mann–Whitney U test was used to compare variables which were not normally distributed. The frequencies of categorical variables were compared by χ^2^ test or Fisher's exact test when appropriate. The Kaplan–Meier method was performed in the patients with LA enlargement at baseline, and the log-rank test was used to assess differences between the groups. Statistical analyses were performed using SPSS software version 22.0 (SPSS Inc., Chicago, IL, USA) and GraphPad Prism 7 (GraphPad Software Inc., San Diego, CA, USA). *P* < 0.05 was considered statistically significant.

## Results

### Baseline Characteristics

We enrolled 2,128 (1,152 males, 976 females) patients diagnosed of HVD with a mean age of 57.5 years in the present study. Among them, 1,532 (72.0%) patients had mitral valve lesion, which was the most common type, followed by aortic valve lesion (1,205 patients, 56.6%) and tricuspid valve lesion (804 patients, 37.8%). There were 2,005 (94.2%) patients who received valve surgery, 1,327 (62.4%) of whom completed the follow-up (Figure 1).

The baseline characteristics of all groups by thyroid function categories are shown in Table 1. Among the study population, 463 (21.8%) patients had hypothyroidism (404 subclinical hypothyroidism patients and 59 overt hypothyroidism patients), and 1,665 (78.2%) patients were euthyroid. As expected, patients of the hypothyroidism group were older and were more likely to be female compared to the euthyroid group. BMI and the proportions of smoking, drinking, prior PCI, and valve surgery were similar. Additionally, the hypothyroidism group had significantly more baseline comorbidities including diabetes mellitus, congestive heart failure, coronary artery disease, atrial flutter/fibrillation, chronic kidney disease, and chronic liver disease.

**Table 1:** Baseline characteristics of the study population.

**Variables**	**All patients**	**Euthyroid group**	**Patients with hypothyroidism**			*P*-value^*^
	**(*n* = 2,128)**	**(*n* = 1,665)**				
			**Hypothyroidism group**	**SCHypoT group**	**OHypoT group**	
			**(*n* = 463)**	**(*n* = 404)**	**(*n* = 59)**	
Age (years)	57.5 ± 12.6	56.9 ± 12.8	59.6 ± 11.5	59.6 ± 11.7	59.5 ± 9.6	**<0.001**
Weight (kg)	63.7 ± 11.5	64.2 ± 11.5	61.7 ± 11.0	62.0 ± 11.2	60.1 ± 10.1	**<0.001**
Height (cm)	164.0 ± 8.6	164.5 ± 8.6	162.2 ± 8.4	162.4 ± 8.5	161.1 ± 7.7	**<0.001**
BMI (kg/m^2^)	23.6 ± 3.2	23.6 ± 3.2	23.4 ± 3.3	23.4 ± 3.4	23.1 ± 2.9	0.136
SBP (mmHg)	126.0 ± 16.1	126.2 ± 15.7	125.3 ± 17.3	125.3 ± 17.2	125.6 ± 18.0	0.350
DBP (mmHg)	74.9 ± 10.1	74.8 ± 10.0	75.4 ± 10.4	75.1 ± 10.3	77.2 ± 10.8	0.313
TSH (mIU/L)	2.54 (1.75–3.91)	2.19 (1.55–2.91)	5.60 (4.70–7.20)	5.36 (4.65–6.44)	13.74 (11.24–17.38)	**<0.001**
Free T_3_ (pmol/L)	4.55 (4.10–5.00)	4.60 (4.20–5.00)	4.40 (3.90–4.80)	4.40 (3.90–4.80)	4.20 (3.70–4.60)	**<0.001**
Free T_4_ (pmol/L)	16.90 (15.30–18.70)	17.10 (15.60–18.80)	16.10 (14.40–18.00)	16.40 (14.63–18.28)	14.40 (12.60–16.50)	**<0.001**
Male (%)	1,152 (54.1)	962 (57.8)	190 (41.0)	170 (42.1)	20 (33.9)	**0.001**
Smoking (%)	338 (15.9)	291 (17.5)	47 (10.2)	38 (9.4)	9 (15.3)	0.581
Drinking (%)	245 (11.5)	207 (12.4)	38 (8.2)	33 (8.2)	5 (8.5)	0.322
Prior PCI (%)	26 (1.2)	16 (1.0)	10 (2.2)	9 (2.2)	1 (1.7)	0.459
**NYHA functional class**						**<0.001**
I/II (%)	902 (42.4)	768 (46.1)	134 (28.9)	124 (30.7)	10 (16.9)	
III/IV (%)	1,226 (57.6)	897 (53.9)	329 (71.1)	280 (69.3)	49 (83.1)	
**Valvular lesion type**						**<0.001**
Single valvular lesion (%)	1,039 (48.8)	879 (52.8)	160 (34.6)	146 (36.1)	14 (23.7)	
Multiple valvular lesion (%)	1,089 (51.2)	786 (47.2)	303 (65.4)	258 (63.9)	45 (76.3)	
**Comorbidities**						
Hypertension (%)	754 (35.4)	589 (35.4)	165 (35.6)	142 (35.1)	23 (39.0)	0.917
Diabetes mellitus (%)	175 (8.2)	126 (7.6)	49 (10.6)	45 (11.1)	4 (6.8)	**0.037**
Congestive heart failure (%)	15 (0.7)	8 (0.5)	7 (1.5)	6 (1.5)	1 (1.7)	**0.019**
Coronary artery disease (%)	210 (9.9)	145 (8.7)	65 (14.0)	59 (14.6)	6 (10.2)	**0.001**
Atrial flutter/fibrillation (%)	718 (33.7)	499 (30.0)	219 (47.3)	184 (45.5)	35 (59.3)	**<0.001**
Cerebrovascular disease (%)	153 (7.2)	111 (6.7)	42 (9.1)	35 (8.7)	7 (11.9)	0.076
Chronic kidney disease (%)	38 (1.8)	22 (1.3)	16 (3.5)	13 (3.2)	3 (5.1)	**0.002**
Chronic liver disease (%)	53 (2.5)	35 (2.1)	18 (3.3)	16 (4.0)	2 (3.4)	**0.029**
**Medications**						
ACEI/ARBs use (%)	296 (13.9)	221 (13.3)	75 (16.2)	65 (16.1)	10 (16.9)	0.108
Statins use (%)	48 (2.3)	36 (2.2)	12 (2.6)	11 (2.7)	1 (1.7)	0.582
ARD (mm)	34.2 ± 5.7	34.5 ± 5.7	33.1 ± 5.4	33.3 ± 5.5	31.9 ± 4.7	**<0.001**
LAD (mm)	48.5 ± 9.9	48.0 ± 9.9	50.3 ± 9.6	50.0 ± 9.5	52.8 ± 10.0	**<0.001**
LVEDD (mm)	54.3 ± 9.1	54.6 ± 9.0	53.2 ± 9.6	53.3 ± 9.7	52.4 ± 8.5	**0.002**
LVESD (mm)	36.4 ± 8.7	36.6 ± 8.5	35.8 ± 9.1	36.0 ± 9.3	34.9 ± 7.6	0.095
IVS (mm)	10.3 ± 2.0	10.3 ± 2.0	10.3 ± 2.2	10.3 ± 2.3	10.2 ± 2.0	0.959
LVPW (mm)	9.9 ± 1.7	9.9 ± 1.6	9.9 ± 1.9	9.9 ± 1.9	9.9 ± 1.7	0.392
PASP (mmHg)	42.8 ± 13.7	42.4 ± 13.6	44.3 ± 13.8	44.1 ± 13.8	46.1 ± 14.0	**0.008**
LVEF (%)	61.0 ± 8.9	61.2 ± 8.8	60.5 ± 9.3	60.5 ± 9.2	60.5 ± 9.9	0.150
Surgery (%)	2,005 (94.2)	1,573 (94.5)	432 (93.3)	374 (92.6)	58 (98.3)	0.340
**Surgery type**						**<0.001**
Single valve surgery (%)	1,155 (57.6)	951(60.5)	204 (47.2)	180 (48.1)	24 (41.4)	
Multiple valve surgery (%)	849 (42.4)	621 (39.5)	228 (52.8)	194 (51.9)	34 (58.6)	
ICU length of stay (days)	2.2 ± 2.5	2.1 ± 2.3	2.6 ± 3.2	2.5 ± 2.8	3.3 ± 4.8	**<0.001**
ICU reentry (%)	12 (1.2)	11 (0.7)	13 (3.0)	10 (2.7)	3 (5.2)	**<0.001**
Hospital length of stay (days)	9.8 ± 4.9	9.4 ± 4.3	11.3 ± 6.6	11.2 ± 5.4	11.9 ± 11.6	**<0.001**
Number of follow-up patients	1,327 (66.2)	1,042 (66.2)	285 (66.0)	245 (65.6)	40 (69.0)	0.916
Follow-up period (months)	6.7 ± 4.0	6.6 ± 3.9	7.1 ± 4.1	7.0 ± 4.2	7.7 ± 3.8	0.054

* Comparison between hypothyroidism and euthyroid group.

TSH, FT_3_, and FT_4_ data are expressed as median (IQR), while other data are expressed as mean ± standard deviation or n (%).

SCHypoT, subclinical hypothyroidism; OHypoT, overt hypothyroidism; BMI, body mass index; SBP, systolic blood pressure; DBP, diastolic blood pressure; PCI, percutaneous coronary intervention; ACEI, angiotensin-converting enzyme inhibitors; ARB, angiotensin receptor blockers; NYHA, New York Heart Association; ARD, aortic root diameter; LAD, left atrial diameter; LVEDD, left ventricular end-diastole diameter; LVESD, left ventricular end-systole diameter; IVS, interventricular septum thickness; LVPW, left ventricular posterior wall thickness; PASP, pulmonary artery systolic pressure; LVEF, left ventricular ejection fraction.

*Bold values indicates statistical significant P <0.05*.

### The Clinical and Echocardiographic Characteristics at Baseline: Hypothyroidism Group vs. Euthyroid Group

As shown in Table 2, after 1:2 PS matching (381 cases and 715 controls matched), all the absolute SD were <0.10, representing all demographic and clinical variables which were comparable between SCHypoT and euthyroid groups. In the SCHypoT group, LAD, IVS, LVPW, and hospital length of stay were significantly increased, LVEF was significantly decreased, and the percentage of III/IV NYHA class and multiple valvular lesions was significantly higher compared to the euthyroid group.

**Table 2:** Comparison of SCHypoT and euthyroid group at baseline by PS matching analysis.

**Variables**	**Before PS matching**		**SD**	***P*-value**	**After PS matching (1:2)**		**SD**	***P*-value**
	**Euthyroid group**	**SCHypoT group**			**Euthyroid group**	**SCHypoT group**		
	**(*n* = 1,665)**	**(*n* = 404)**			**(*n* = 715)**	**(*n* = 381)**		
**Thyroid function**								
TSH (mIU/L)	2.19 (1.55–2.91)	5.36 (4.65–6.44)		**<0.001**	2.19 (1.57–3.00)	5.30 (4.64–6.44)		**<0.001**
Free T_3_ (pmol/L)	4.60 (4.20–5.00)	4.40 (3.90–4.80)		**<0.001**	4.50 (4.20–4.90)	4.40 (4.00–4.80)		**<0.001**
Free T_4_ (pmol/L)	17.10 (15.60–18.80)	16.40 (14.63–18.28)		**<0.001**	17.20 (15.60–18.90)	16.40 (14.70–18.30)		**<0.001**
**Variables for matching**								
Age (years)	56.9 ± 12.8	59.6 ± 11.7	0.220	**<0.001**	59.2 ± 11.5	59.2 ± 11.8	0.004	0.954
Weight (kg)	64.2 ± 11.5	62.0 ± 11.2	−0.194	**<0.001**	62.1 ± 10.8	62.0 ± 11.2	−0.005	0.933
Height (cm)	164.5 ± 8.6	162.4 ± 8.5	−0.246	**<0.001**	162.5 ± 8.5	162.4 ± 8.5	−0.005	0.939
Male (%)	962 (57.8)	170 (42.1)	−0.318	**<0.001**	308 (43.1)	162 (42.5)	−0.012	0.859
Smoking (%)	291 (17.5)	38 (9.4)	−0.239	**<0.001**	69 (9.7)	37 (9.7)	0.000	0.974
Drinking (%)	207 (12.4)	33 (8.2)	−0.139	**0.016**	61 (8.5)	33 (8.7)	0.007	0.942
Prior PCI (%)	16 (1.0)	9 (2.2)	0.096	**0.037**	8 (1.1)	5 (1.3)	0.018	0.778
**Comorbidities**								
Hypertension (%)	589 (35.4)	142 (35.1)	−0.006	0.932	251 (35.1)	130 (34.1)	−0.021	0.745
Diabetes mellitus (%)	126 (7.6)	45 (11.1)	0.120	**0.019**	71 (9.9)	42 (11.0)	0.036	0.571
Congestive heart failure (%)	8 (0.5)	6 (1.5)	0.101	**0.027**	4 (0.6)	3 (0.8)	0.024	0.699
Coronary artery disease (%)	145 (8.7)	59 (14.6)	0.185	**<0.001**	84 (11.7)	46 (12.1)	0.012	0.874
Atrial flutter/fibrillation (%)	499 (30.0)	184 (45.5)	0.324	**<0.001**	290 (40.6)	165 (43.3)	0.055	0.379
Cerebrovascular disease (%)	111 (6.7)	35 (8.7)	0.075	0.160	58 (8.1)	29 (7.6)	−0.019	0.770
Chronic kidney disease (%)	22 (1.3)	13 (3.2)	0.128	**0.008**	10 (1.4)	6 (1.6)	0.016	0.817
Chronic liver disease (%)	35 (2.1)	16 (4.0)	0.111	**0.031**	15 (2.1)	14 (3.7)	0.095	0.121
**Medications**								
ACEI/ARBs use (%)	221 (13.3)	65 (16.1)	0.079	0.141	107 (15.0)	55 (14.4)	−0.017	0.814
Statins use (%)	36 (2.2)	11 (2.7)	0.032	0.498	19 (2.7)	11 (2.9)	0.012	0.824
**Variables for comparison**								
ARD (mm)	34.5 ± 5.7	33.3 ± 5.5		**<0.001**	33.5 ± 5.2	33.4 ± 5.6		0.691
LAD (mm)	48.0 ± 9.9	50.0 ± 9.5		**<0.001**	48.6 ± 9.8	49.9 ± 9.5		**0.031**
LVEDD (mm)	54.6 ± 9.0	53.3 ± 9.7		**0.007**	52.8 ± 8.6	53.5 ± 9.7		0.174
LVESD (mm)	36.6 ± 8.5	36.0 ± 9.3		0.199	35.1 ± 7.8	36.1 ± 9.4		0.053
IVS (mm)	10.3 ± 2.0	10.3 ± 2.3		0.817	10.1 ± 1.9	10.3 ± 2.3		**0.047**
LVPW (mm)	9.9 ± 1.6	9.9 ± 1.9		0.361	9.7 ± 1.6	10.0 ± 2.0		**0.011**
PASP (mmHg)	42.4 ± 13.6	44.1 ± 13.8		**0.029**	43.1 ± 14.2	44.1 ± 13.9		0.240
LVEF (%)	61.2 ± 8.8	60.5 ± 9.2		0.175	61.6 ± 8.2	60.6 ± 9.0		**0.040**
**NYHA functional class**				**<0.001**				**<0.001**
I/II (%)	768 (46.1)	124 (30.7)			314 (43.9)	122 (32.0)		
III/IV (%)	897 (53.9)	280 (69.3)			401 (56.1)	259 (68.0)		
**Valvular lesion type**				**<0.001**				**<0.001**
Single valvular lesion (%)	879 (52.8)	146 (36.1)			340 (47.6)	136 (35.7)		
Multiple valvular lesion (%)	786 (47.2)	258 (63.9)			375 (52.4)	245 (64.3)		
ICU length of stay (days)	2.1 ± 2.3	2.5 ± 2.8		**0.003**	2.0 ± 2.2	2.2 ± 2.5		0.101
ICU reentry (%)	11 (0.7)	10 (2.7)		**0.001**	5 (0.7)	9 (2.4)		**0.020**
Hospital length of stay (days)	9.4 ± 4.3	11.2 ± 5.4		**<0.001**	8.8 ± 4.9	10.4 ± 5.9		**<0.001**

TSH, FT_3_, and FT_4_ data are expressed as median (IQR), while other data are expressed as mean ± standard deviation or n (%).

SCHypoT, subclinical hypothyroidism; PCI, percutaneous coronary intervention; ACEI, angiotensin-converting enzyme inhibitors; ARB, angiotensin receptor blockers; NYHA, New York Heart Association; ARD, aortic root diameter; LAD, left atrial diameter; LVEDD, left ventricular end-diastole diameter; LVESD, left ventricular end-systole diameter; IVS, interventricular septum thickness; LVPW, left ventricular posterior wall thickness; PASP, pulmonary artery systolic pressure; LVEF, left ventricular ejection fraction; PS, propensity score; SD, standardized differences.

*Bold values indicates statistical significant P <0.05*.

As for the OHypoT group, we performed 1:4 PS matching, 55 cases and 203 controls were matched, and most covariates were balanced between groups except for chronic kidney disease and history of ACEI/ARBs. After PS matching, only LAD was significantly higher among the echocardiographic parameters. Besides, hospital length of stay and proportion of III/IV NYHA class of the OHypoT group were significantly higher than euthyroid group (Table 3).

**Table 3:** Comparison of OHypoT and euthyroid group at baseline by PS matching analysis.

**Variables**	**Before PS matching**		**SD**	***P*-value**	**After PS matching (1:4)**		**SD**	***P*-value**
	**Euthyroid group**	**OHypoT group**			**Euthyroid group**	**OHypoT group**		
	**(*n* = 1,665)**	**(*n* = 59)**			**(*n* = 203)**	**(*n* = 55)**		
**Thyroid function**								
TSH (mIU/L)	2.19 (1.55–2.91)	13.74 (11.24–17.38)		**<0.001**	2.28 (1.60–3.08)	13.57 (11.14–16.06)		**<0.001**
Free T_3_ (pmol/L)	4.60 (4.20–5.00)	4.20 (3.70–4.60)		**<0.001**	4.40 (4.10–4.90)	4.20 (3.70–4.70)		**0.012**
Free T_4_ (pmol/L)	17.10 (15.60–18.80)	14.40 (12.60–16.50)		**<0.001**	17.70 (16.00–19.10)	14.60 (12.70–16.50)		**<0.001**
**Variables for matching**								
Age (years)	56.9 ± 12.8	59.5 ± 9.6	0.230	0.233	59.4 ± 12.1	59.4 ± 9.8	0.002	0.638
Weight (Kg)	64.2 ± 11.5	60.1 ± 10.1	−0.379	**0.003**	60.2 ± 10.8	60.1 ± 10.3	−0.009	0.831
Height (cm)	164.5 ± 8.6	161.1 ± 7.7	−0.417	**0.002**	161.2 ± 8.6	161.3 ± 7.8	0.013	0.861
Male (%)	962 (57.8)	20 (33.9)	−0.494	**<0.001**	75 (36.9)	20 (36.4)	−0.010	0.937
Smoking (%)	291 (17.5)	9 (15.3)	−0.059	0.658	39 (19.2)	9 (16.4)	−0.073	0.630
Drinking (%)	207 (12.4)	5 (8.5)	−0.128	0.363	22 (10.8)	5 (9.1)	−0.057	0.707
Prior PCI (%)	16 (1.0)	1 (1.7)	0.061	0.488	2 (1.0)	1 (1.8)	0.068	0.514
**Comorbidities**								
Hypertension (%)	589 (35.4)	23 (39.0)	0.075	0.569	73 (36.0)	20 (36.4)	−0.010	0.956
Diabetes mellitus (%)	126 (7.6)	4 (6.8)	−0.031	1.000	16 (7.9)	4 (7.3)	−0.023	1.000
Congestive heart failure (%)	8 (0.5)	1 (1.7)	0.115	0.270	0 (0.0)	0 (0.0)		
Coronary artery disease (%)	145 (8.7)	6 (10.2)	0.051	0.696	19 (9.4)	5 (9.1)	−0.010	0.951
Atrial flutter/fibrillation (%)	499 (30.0)	35 (59.3)	0.617	**<0.001**	108 (53.2)	31 (56.4)	0.064	0.677
Cerebrovascular disease (%)	111 (6.7)	7 (11.9)	0.180	0.120	17 (8.4)	4 (7.3)	−0.041	1.000
Chronic kidney disease (%)	22 (1.3)	3 (5.1)	0.217	0.051	5 (2.5)	3 (5.5)	0.154	0.373
Chronic liver disease (%)	35 (2.1)	2 (3.4)	0.080	0.364	6 (3.0)	2 (3.6)	0.034	0.680
**Medications**								
ACEI/ARBs use(%)	221 (13.3)	65 (16.1)	0.079	0.415	33 (16.3)	7 (12.7)	−0.102	0.521
Statins use(%)	36 (2.2)	11 (2.7)	0.032	1.000	4 (2.0)	1 (1.8)	−0.015	1.000
**Variables for comparison**								
ARD (mm)	34.5 ± 5.7	31.9 ± 4.7		**<0.001**	33.0 ± 5.3	32.0 ± 4.7		0.215
LAD (mm)	48.0 ± 9.9	52.8 ± 10.0		**<0.001**	49.3 ± 8.5	52.9 ± 10.4		**0.008**
LVEDD (mm)	54.6 ± 9.0	52.4 ± 8.5		0.055	52.4 ± 9.1	52.8 ± 8.5		0.795
LVESD (mm)	36.6 ± 8.5	34.9 ± 7.6		0.125	35.1 ± 8.5	35.1 ± 7.7		0.984
IVS (mm)	10.3 ± 2.0	10.2 ± 2.0		0.604	9.9 ± 2.0	10.2 ± 2.0		0.473
LVPW (mm)	9.9 ± 1.6	9.9 ± 1.7		0.966	9.6 ± 1.7	9.9 ± 1.7		0.368
PASP (mmHg)	42.4 ± 13.6	46.1 ± 14.0		**0.039**	45.2 ± 13.1	46.6 ± 14.4		0.502
LVEF (%)	61.2 ± 8.8	60.5 ± 9.9		0.540	60.9 ± 9.1	60.4 ± 10.0		0.691
**NYHA functional class**				**<0.001**				**0.001**
I/II (%)	768 (46.1)	10 (16.9)			77 (37.9)	8 (14.5)		
III/IV (%)	897 (53.9)	49 (83.1)			126 (62.1)	47 (85.5)		
**Valvular lesion type**				**<0.001**				0.183
Single valvular lesion (%)	879 (52.8)	14 (23.7)			71 (25.0)	14 (25.5)		
Multiple valvular lesion (%)	786 (47.2)	45 (76.3)			132 (65.0)	41 (74.5)		
ICU length of stay (days)	2.1 ± 2.3	3.3 ± 4.8		**<0.001**	2.5 ± 3.6	3.3 ± 4.9		0.235
ICU re-etry (%)	11 (0.7)	3 (5.2)		**0.012**	4 (2.1)	3 (5.6)		0.184
Hospital length of stay (days)	9.4 ± 4.3	11.9 ± 11.6		**<0.001**	9.7 ± 4.5	12.1 ± 12.0		**0.027**

TSH, FT_3_, and FT_4_ data are expressed as median (IQR), while other data are expressed as mean ± standard deviation or n (%).

OHypoT, overt hypothyroidism; PCI, percutaneous coronary intervention; ACEI, angiotensin-converting enzyme inhibitors; ARB, angiotensin receptor blockers; NYHA, New York Heart Association; ARD, aortic root diameter; LAD, left atrial diameter; LVEDD, left ventricular end-diastole diameter; LVESD, left ventricular end-systole diameter; IVS, interventricular septum thickness; LVPW, left ventricular posterior wall thickness; PASP, pulmonary artery systolic pressure; LVEF, left ventricular ejection fraction; PS, propensity score; SD, standardized difference.

*Bold values indicates statistical significant P <0.05*.

### The Echocardiographic Characteristics After Valve Surgery: Hypothyroidism Group vs. Euthyroid Group

A total of 2,005 (94.2%) patients underwent heart valve surgery, and 1,327 (66.2%) patients (285 hypothyroidism patients and 1,042 euthyroid patients, Table 1) completed the follow-up and had the echocardiographic data. In the SCHypoT and OHypoT groups, the follow-up echocardiographic data of 245 and 40 patients were recorded, respectively (Table 1).

After 1:2 PS matching for the SCHypoT group, 221 cases and 408 controls were matched with all covariates well-balanced. Compared to the euthyroid group, the value of LAD was significantly higher in the SCHypoT group after valve surgery (*P* = 0.048, Table 4). In the 1:4 PS matching for the OHypoT group, 35 cases and 108 controls were matched, while age, hypertension, diabetes mellitus, cerebrovascular disease, chronic kidney disease, and history of smoking were unmatched between groups. Although the LAD in the OHypoT group was higher than that in the euthyroid group, the difference was not statistically significant (Table 5).

**Table 4:** Comparison of SCHypoT and euthyroid group after surgery by PS matching analysis.

**Variables**	**Before PS matching**		**SD**	***P*-value**	**After PS matching (1:2)**		**SD**	***P*-value**
	**Euthyroid group**	**SCHypoT group**			**Euthyroid group**	**SCHypoT group**		
	**(*n* = 1,042)**	**(*n* = 245)**			**(*n* = 408)**	**(*n* = 221)**		
**Thyroid function**								
TSH (mIU/L)	2.23 (1.56–2.92)	5.41 (4.66–6.45)		**<0.001**	2.28 (1.60–2.99)	5.50 (4.66–6.51)		**<0.001**
Free T_3_ (pmol/L)	4.60 (4.20–5.00)	4.50 4.10–4.90)		**<0.001**	4.50 (4.10–5.00)	4.50 (4.10–4.90)		**0.007**
Free T_4_ (pmol/L)	17.10 (15.70–18.70)	16.30 (14.70–18.20)		**<0.001**	17.00 (15.70–18.70)	16.20 (14.75–18.10)		**<0.001**
**Variables for matching**								
Age (years)	56.1 ± 12.7	59.1 ± 12.1	0.242	**0.001**	58.1 ± 11.5	58.2 ± 11.7	0.010	0.897
Weight (Kg)	64.6 ± 11.5	62.4 ± 11.1	−0.195	**0.006**	62.0 ± 11.5	62.5 ± 11.0	0.053	0.531
Height (cm)	164.9 ± 8.6	162.5 ± 8.3	−0.284	**<0.001**	162.7 ± 8.8	162.9 ± 8.1	0.026	0.766
Male (%)	599 (57.5)	99 (40.4)	−0.347	**<0.001**	177 (43.4)	95 (43.0)	−0.008	0.924
Smoking (%)	170 (16.3)	22 (9.0)	−0.221	**0.004**	36 (8.8)	22(10.0)	0.041	0.640
Drinking (%)	120 (11.5)	19 (7.8)	−0.126	0.088	34 (8.3)	19 (8.6)	0.011	0.909
Prior PCI (%)	10 (1.0)	4 (1.6)	0.053	0.320	3 (0.7)	3 (1.4)	0.069	0.443
**Comorbidities**								
Hypertension (%)	363 (34.8)	76 (31.0)	−0.081	0.257	117 (28.7)	69 (31.2)	0.055	0.504
Diabetes mellitus (%)	71 (6.8)	26 (10.6)	0.135	**0.043**	28 (6.9)	19 (8.6)	0.064	0.430
Congestive heart failure (%)	5 (0.5)	1 (0.4)	−0.015	1.000	1 (0.2)	1 (0.5)	0.051	1.000
Coronary artery disease (%)	85 (8.2)	35 (14.3)	0.194	**0.003**	37 (9.1)	25 (11.3)	0.073	0.367
Atrial flutter/fibrillation (%)	294 (28.2)	104 (42.4)	0.300	**<0.001**	151 (37.0)	89 (40.3)	0.068	0.421
Cerebrovascular disease (%)	59 (5.7)	18 (7.7)	0.080	0.317	25 (6.1)	16 (7.2)	0.044	0.590
Chronic kidney disease (%)	10 (1.0)	6 (2.4)	0.108	0.058	4 (1.0)	4 (1.8)	0.068	0.375
Chronic liver disease (%)	20 (1.9)	11 (4.6)	0.153	**0.018**	11 (2.7)	7 (3.2)	0.030	0.735
**Medications**								
ACEI/ARBs use (%)	137 (13.1)	41 (16.7)	0.101	0.143	50 (12.3)	32 (14.5)	0.065	0.429
Statins use (%)	23 (2.2)	7 (2.9)	0.044	0.544	9 (2.2)	7 (3.2)	0.062	0.465
**NYHA functional class**			0.220	**0.002**			0.002	0.989
I/II (%)	491 (47.1)	89 (36.3)			159 (39.0)	86 (38.9)		
III/IV (%)	551 (52.9)	156 (63.7)			249 (61.0)	135 (61.1)		
**Surgery Type**			0.205	**0.004**			0.030	0.724
Single valve surgery (%)	649 (62.3)	128 (52.2)			222 (54.4)	117 (52.9)		
Multiple valve surgery (%)	393 (37.7)	117 (47.8)			186 (45.6)	104 (47.1)		
Follow-up period (months)	6.6 ± 3.9	7.0 ± 4.2	0.099	0.140	6.9 ± 4.0	7.0 ± 4.1	0.025	0.822
**Variables for comparison**								
ARD (mm)	33.9 ± 10.3	32.8 ± 4.3		**0.009**	33.2 ± 4.147	33.0 ± 4.4		0.591
LAD (mm)	43.4 ± 7.5	45.5 ± 7.6		**<0.001**	44.0 ± 7.9	45.2 ± 7.5		**0.048**
LVEDD (mm)	48.0 ± 5.7	48.0 ± 6.2		0.978	47.4 ± 6.1	48.1 ± 6.3		0.142
LVESD (mm)	31.7 ± 5.5	31.7 ± 6.2		0.990	31.4 ± 5.6	31.8 ± 6.4		0.346
IVS (mm)	10.0 ± 1.8	10.0 ± 2.0		0.869	9.9 ± 1.7	10.1 ± 2.0		0.208
LVPW (mm)	9.6 ± 1.4	9.6 ± 1.6		0.931	9.5 ± 1.3	9.6 ± 1.6		0.391
PASP (mmHg)	33.4 ± 5.9	34.0 ± 6.3		0.127	33.5 ± 5.9	33.9 ± 6.3		0.362
LVEF (%)	62.5 ± 7.1	62.3 ± 7.2		0.764	62.3 ± 7.3	62.2 ± 7.4		0.830

TSH, FT_3_, and FT_4_ data are expressed as median (IQR), while other data are expressed as mean ± standard deviation or n (%).

SCHypoT, subclinical hypothyroidism; PCI, percutaneous coronary intervention; ACEI, angiotensin-converting enzyme inhibitors; ARB, angiotensin receptor blockers; NYHA, New York Heart Association; ARD, aortic root diameter; LAD, left atrial diameter; LVEDD, left ventricular end-diastole diameter; LVESD, left ventricular end-systole diameter; IVS, interventricular septum thickness; LVPW, left ventricular posterior wall thickness; PASP, pulmonary artery systolic pressure; LVEF, left ventricular ejection fraction; PS, propensity score; SD, standardized differences.

*Bold values indicates statistical significant P < 0.05*.

**Table 5:** Comparison of OHypoT and euthyroid group after surgery by PS matching analysis.

**Variables**	**Before PS matching**		**SD**	***P*-value**	**After PS matching (1:4)**		**SD**	***P*-value**
	**Euthyroid group**	**OHypoT group**			**Euthyroid group**	**OHypoT group**		
	**(*n* = 1,042)**	**(*n* = 40)**			**(*n* = 108)**	**(*n* = 35)**		
**Thyroid function**								
TSH (mIU/L)	2.23 (1.56–2.92)	13.44 (10.57–16.31)		**<0.001**	2.22 (1.59–2.95)	13.53 (10.52–16.40)		**<0.001**
Free T_3_ (pmol/L)	4.60 (4.20–5.00)	4.15 (3.70–4.60)		**<0.001**	4.60 (4.10–4.90)	4.20 (3.70–4.60)		**<0.001**
Free T_4_ (pmol/L)	17.10 (15.70–18.70)	14.35 (12.48–16.58)		**<0.001**	17.20 (15.90–18.80)	14.60 (12.70–16.60)		**<0.001**
**Variables for matching**								
Age (years)	56.1 ± 12.7	59.9 ± 10.0	0.332	0.099	58.9 ± 11.3	59.9 ± 10.7	0.105	0.195
Weight (Kg)	64.6 ± 11.5	60.3 ± 10.8	−0.385	**0.010**	60.3 ± 10.3	60.5 ± 11.2	0.020	0.916
Height (cm)	164.9 ± 8.6	161.6 ± 8.4	−0.388	**0.017**	161.2 ± 7.8	161.7 ± 8.3	0.062	0.755
Male (%)	599 (57.5)	14 (35.0)	−0.463	**0.005**	36 (33.3)	12 (34.3)	0.021	0.917
Smoking (%)	170 (16.3)	6 (15.0)	−0.036	0.825	8 (7.4)	5 (14.3)	0.223	0.219
Drinking (%)	120 (11.5)	2 (5.0)	−0.238	0.305	5 (4.6)	2 (5.7)	0.050	0.680
Prior PCI (%)	10 (1.0)	1 (2.5)	0.115	0.341	2 (1.9)	1 (2.9)	0.065	0.572
**Comorbidities**								
Hypertension (%)	363 (34.8)	16 (40.0)	0.108	0.502	35 (32.4)	14 (40.0)	0.159	0.411
Diabetes mellitus (%)	71 (6.8)	3 (7.5)	0.027	0.750	2 (1.9)	2 (5.7)	0.200	0.251
Congestive heart failure (%)	5 (0.5)	0 (0.0)	−0.100	1.000	0 (0.0)	0 (0.0)		
Coronary artery disease (%)	85 (8.2)	4 (10.0)	0.063	0.564	7 (6.5)	3 (8.6)	0.080	0.707
Atrial flutter/fibrillation (%)	294 (28.2)	25 (62.5)	0.734	**<0.001**	60 (55.6)	21 (60.0)	0.089	0.645
Cerebrovascular disease (%)	59 (5.7)	4 (10.0)	0.160	0.286	9 (8.3)	2 (5.7)	−0.102	0.590
Chronic kidney disease (%)	10 (1.0)	2 (5.0)	0.236	0.070	0 (0.0)	1 (2.9)	0.244	0.245
Chronic liver disease (%)	20 (1.9)	2 (5.0)	0.170	0.194	4 (3.7)	2 (5.7)	0.095	0.635
**Medications**								
ACEI/ARBs use (%)	137 (13.1)	7 (17.5)	0.113	0.426	19 (17.6)	6 (17.1)	−0.013	0.951
Statins use (%)	23 (2.2)	0 (0.0)	−0.212	1.000	0 (0.0)	0 (0.0)		
**NYHA functional class**			0.740	**<0.001**			−0.011	0.948
I/II (%)	491 (47.1)	6 (15.0)			18 (16.7)	6 (17.1)		
III/IV (%)	551 (52.9)	34 (85.0)			90 (83.3)	29 (82.9)		
**Surgery Type**			0.458	**0.004**			0.030	0.869
Single valve surgery (%)	649 (62.3)	16 (40.0)			48 (44.4)	15 (42.9)		
Multiple valve surgery (%)	393 (37.7)	24 (60.0)			60 (55.6)	20 (57.1)		
Follow-up period (months)	6.6 ± 3.9	7.7 ± 3.8	0.286	**0.024**	7.1 ± 4.0	7.3 ± 3.7	0.052	0.822
**Variables for comparison**								
ARD (mm)	33.9 ± 10.3	32.4 ± 4.5		0.367	32.7 ± 4.3	32.3 ± 4.6		0.636
LAD (mm)	43.4 ± 7.5	48.5 ± 10.1		**0.003**	44.7 ± 8.0	46.9 ± 6.9		0.134
LVEDD (mm)	48.0 ± 5.8	48.2 ± 5.4		0.896	47.3 ± 5.8	47.8 ± 5.7		0.643
LVESD (mm)	31.7 ± 5.5	32.0 ± 5.3		0.801	31.1 ± 5.2	31.9 ± 5.6		0.452
IVS (mm)	10.0 ± 1.9	9.3 ± 1.8		**0.016**	9.6 ± 1.6	9.4 ± 1.8		0.400
LVPW (mm)	9.6 ± 1.4	8.8 ± 1.2		**0.001**	9.3 ± 1.4	8.9 ± 1.2		0.080
PASP (mmHg)	33.4 ± 5.9	33.5 ± 6.4		0.958	35.1 ± 7.5	33.8 ± 6.7		0.329
LVEF (%)	62.5 ± 7.1	60.9 ± 7.0		0.181	62.6 ± 6.5	60.6 ± 7.2		0.128

TSH, FT_3_, and FT_4_ data are expressed as median (IQR), while other data are expressed as mean ± standard deviation or n (%).

OHypoT, overt hypothyroidism; PCI, percutaneous coronary intervention; ACEI, angiotensin-converting enzyme inhibitors; ARB, angiotensin receptor blockers; NYHA, New York Heart Association; ARD, aortic root diameter; LAD, left atrial diameter; LVEDD, left ventricular end-diastole diameter; LVESD, left ventricular end-systole diameter; IVS, interventricular septum thickness; LVPW, left ventricular posterior wall thickness; PASP, pulmonary artery systolic pressure; LVEF, left ventricular ejection fraction; PS, propensity score; SD, standardized differences.

*Bold values indicate statistical significance at P < 0.05*.

### Recovery of LA Enlargement After Valve Surgery: Hypothyroidism Group vs. Euthyroid Group

In the patients who completed the follow-up, a total of 1,079 (94.2%) patients were identified as LA enlargement (LAD > 40 mm) before valve surgery, of whom 245 patients had hypothyroidism and 834 patients were euthyroid. The SCHypoT group and OHypoT group had 207 and 28 patients with preoperative LA enlargement, respectively.

**Figure 2:**
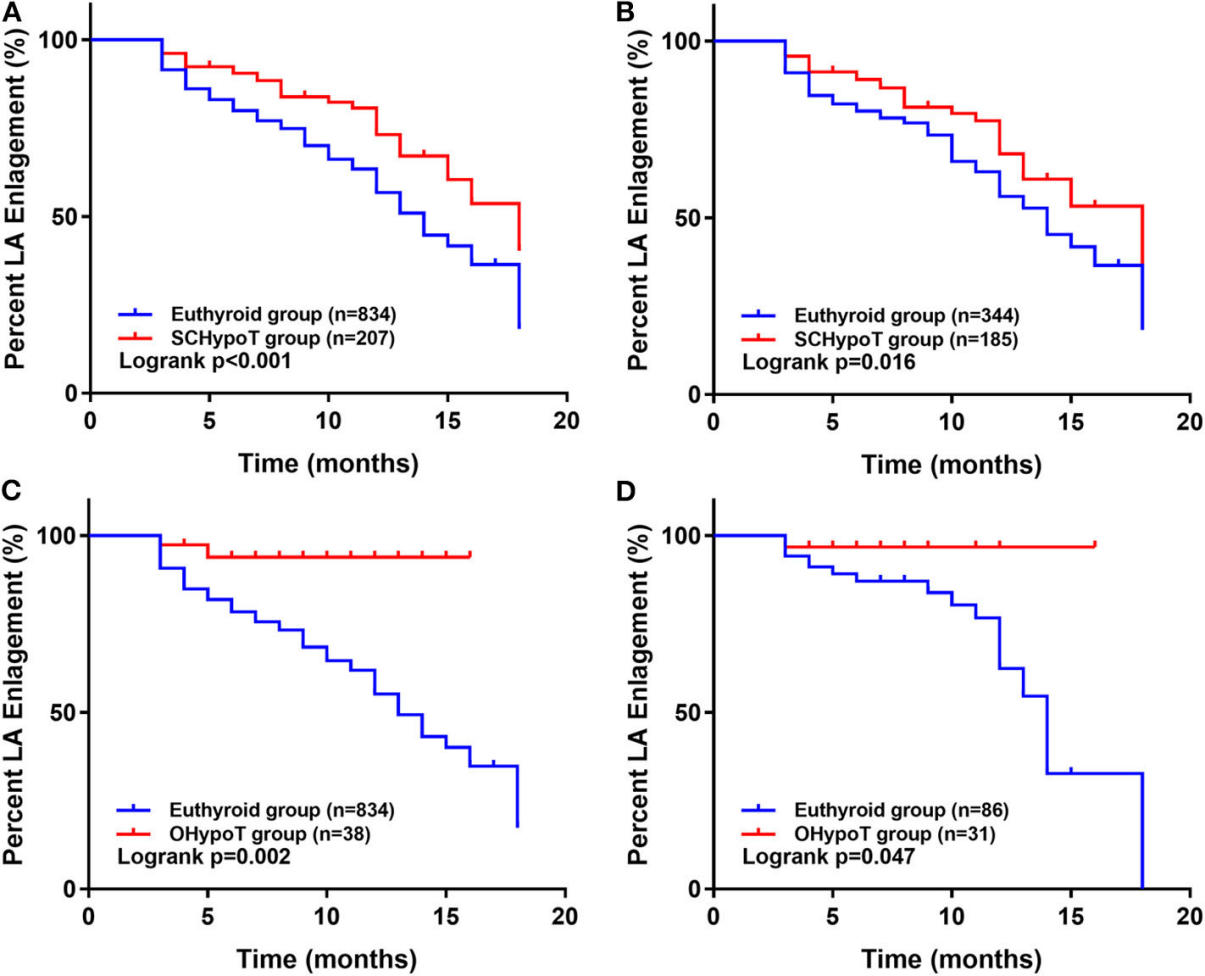
Kaplan–Meier analysis of recovery of LA enlargement after surgery in the hypothyroidism group and euthyroid group. The unmatched **(A)** and propensity score-matched **(B)** analysis in the SCHypoT and euthyroid groups. The unmatched **(C)** and propensity score-matched **(D)** analysis in the OHypoT and euthyroid groups. LA, left atrium; SCHypoT, subclinical hypothyroidism; OHypoT, overt hypothyroidism.

The 1:2 PS matching for the SCHypoT group is shown in Supplementary Table 1, and all covariates were well balanced. Compared to the euthyroid group, the recovery rates of LA enlargement were significantly lower in the SCHypoT group (before PS matching: log-rank *P* < 0.001; after PS matching: log-rank *P* = 0.016, Figures 2A,B). The 1:4 PS matching for the OHypoT group is shown in Supplementary Table 2, and the SDs of height, sex, chronic liver disease, and history of drinking were higher than 0.10. Compared to the euthyroid group, the OHypoT group had a significantly lower recovery rate of LA enlargement (before PS matching: log-rank *P* = 0.002; after PS matching: log-rank *P* = 0.047, Figures 2C,D). These results indicated that hypothyroidism has a negative impact on the recovery of LA enlargement in patients after valve surgery.

## Discussion

Our study is the first study which examined the influence of hypothyroidism on patients with HVD. The main finding of our study is that hypothyroidism was associated with larger LAD in patients with HVD, which may suggest that hypothyroidism is a risk factor of LA enlargement of HVD. At baseline, hypothyroidism was associated with significantly higher LAD, IVS, LVPW, and PASP; lower LVEF; poorer cardiac function; more multiple valvular lesion; and longer ICU and hospital length of stay. After valve surgery, patients with hypothyroidism still had significantly higher LAD, while there are no significant differences in other echocardiographic parameters. Additionally, in the patients with preoperative LA enlargement, hypothyroidism was associated with a significantly lower recovery rate of LA enlargement after valve surgery.

A few previous studies had retrospectively evaluated the effects of hypothyroidism on LAD, but the results were inconsistent. The first was performed in Turkey in 2012, where Ozturk et al. (14) investigated 40 patients with newly diagnosed SCHypoT and another 40 healthy controls and found no difference of LAD between groups. In 2013, Karabag et al. (16) found similar results. Meanwhile, studies performed by Tadic et al. (15, 17–19) in Serbia examined echocardiographic parameters in dozens of women with SCHypoT and healthy control women, and all the results showed no difference in LAD. Moreover, levothyroxine treatment of 1 year for the SCHypoT group failed to recover the LAD significantly (15, 17–19). More recently, Li et al. compared LAD in Chinese DCM patients with or without SCHypoT and found significantly larger LAD in SCHypoT group. The latest literature in 2019 by Dereli et al. (20) studied 40 SCHypoT patients and 40 age- and gender-matched controls, which determined a higher LAD in SCHypoT with borderline insignificant *P*-value (*P* = 0.060). The discrepancy between previous studies and our study may be due to the different study populations and small samples of the previous studies. In our study, the statistically significant difference of LAD between OHypoT and euthyroid groups after surgery disappeared. The small number of patients with OHypoT caused by loss to follow-up which resulted in imperfect PS matching effect precluded precise estimate for this group. Another reason is that we are not fully aware of whether these patients have received levothyroxine treatment after surgery. Among the patients with OHypoT, 21 patients were lost, 32 patients did not receive levothyroxine treatment, and six patients have received levothyroxine treatment after surgery. Levothyroxine treatment may partially restore the enlarged LAD and reduce the difference between OHypoT and euthyroid groups after surgery.

An important target of thyroid hormones is the myocardial interstitium, and normal thyroid function guarantees the normal cardiac structure and mechanical functions (27, 28). Important alterations including increased extracellular collagen and diffuse interstitial space expansion in the myocardial interstitium, which is caused by the development of fibrosis and accumulation of mucopolysaccharide substance, are the main characteristics of hypothyroidism (29–31). Currently, atrial remodeling is commonly estimated by LA size measurement, which has been used by some authors to show atrial function (32, 33). Consistently, in our study, LA remodeling as represented by increased LAD was associated with hypothyroidism in patients with HVD.

We also observed significantly higher IVS and LVPW and lower LVEF in the hypothyroidism group at baseline, which is consistent with previous studies (16–18). Hypothyroidism is supposed to induce increased vascular resistance (34, 35), and significantly higher PASP was found in the hypothyroidism group before surgery in the present study. This observation was in accordance with the study of Zuhur et al., which showed a trend toward higher proportions of mild pulmonary hypertension in hypothyroidism patients compared to euthyroid controls (36).

Our study identified a significant association between hypothyroidism and preoperative AF in patients with HVD, and its proportions were higher in the OHypoT group (59.3%) compared to the SCHypoT group (45.5%) and euthyroid group (30.0%). Atrial enlargement may occur as a consequence of AF (37, 38), and LA enlargement preceding AF or higher LAD may lead to an increased risk of incidence and recurrence of AF (39–42). Additionally, overt and subclinical hypothyroidism has been demonstrated to increase the risk of postoperative AF in the patients underwent cardiac surgery (43–45). However, our study matched AF in the PS analyses, which indicated that the difference of LAD between hypothyroidism and euthyroid groups was independent of AF.

The prevalence of hypothyroidism in the total study population was 21.8% (463/2,128) and was 18.9 and 2.8% for SCHypoT and OHypoT, respectively. This number is higher than the general population in China reported by a recent research, which observed the incidence rate of 16.7% of SCHypoT and 1.11% of OHypoT in 10 cities (46). Actually, hypothyroidism was demonstrated to be associated with several disorders of the cardiovascular system (2–4). These findings showed that hypothyroidism may be a risk factor for HVD.

According to the medical records, there were 11 and nine patients who underwent thyroidectomy in the SCHypoT and OHypoT groups, respectively. However, for the rest of this study population, the etiology and duration of preoperative hypothyroidism were unclear. Several other reasons such as autoimmunity diseases and iodine deficiency may cause thyroid dysfunction, and their effect *per se* on cardiovascular system still needs further investigation. Besides, there is still controversy about whether patients with SCHypoT should be treated. From a cardiac perspective, treatment may reduce the cardiovascular risk and improve cardiac outcomes (47–49). Therefore, thyroid function should be assessed in HVD patients, and randomized controlled trials are needed to evaluate the benefits of levothyroxine treatment in HVD patients with hypothyroidism.

## Strengths and Limitations

To our knowledge, this is the first study to evaluate the influence of hypothyroidism on patients with HVD. We used PS matching to reduce bias. Moreover, we performed analyses before and after surgery to ensure the effect of hypothyroidism on echocardiographic parameters. Our study also has some limitations. First, although PS matching was used to eliminate the differences of covariates between groups, some unknown confounders may still exist. Second, some unbalanced covariates probably due to the small sample size existed in the PS matching for the OHypoT group. Third, the etiology and duration of preoperative hypothyroidism were unclear, and we did not have the information about thyroid function after surgery. For patients of SCHypoT, follow-up is regularly recommended instead of levothyroxine treatment. In the OHypoT group, 21 patients were lost, 32 patients did not receive levothyroxine treatment, and six patients have received levothyroxine treatment after surgery. Therefore, the effect of postoperative levothyroxine treatment cannot be excluded at least in the OHypoT group. Finally, our study focused on the effect of hypothyroidism on cardiac structure, but not the mechanical and electromechanical function.

## Conclusions

In conclusion, hypothyroidism was associated with a larger LAD in patients with HVD before and after surgery, which may suggest that hypothyroidism is a risk factor of LA enlargement of HVD. Besides, hypothyroidism was associated with a significantly lower recovery rate of LA enlargement after valve surgery.

## Data Availability Statement

All datasets generated for this study are included in the article/Supplementary Material.

## Ethics Statement

The studies involving human participants were reviewed and approved by Ethics committee of Zhongshan Hospital of Fudan University. The patients/participants provided their written informed consent to participate in this study.

## Author Contributions

TZ and YL conceived and designed the study. TZ and ZC collected the data. TZ, JZ, and YL analyzed and interpreted the data and wrote the article. All authors contributed to the article and approved the submitted version.

## Conflict of Interest

The authors declare that the research was conducted in the absence of any commercial or financial relationships that could be construed as a potential conflict of interest.
