# Supplementary material for: Impact of Hypothyroidism on Echocardiographic Characteristics of Patients With Heart Valve Disease: A Single-Center Propensity Score-Based Study
Source: Front Endocrinol (Lausanne). 2020 Sep 24;11:554762. doi: 10.3389/fendo.2020.554762 (PMC7542235; doi:10.3389/fendo.2020.554762)
Supplement: Supplementary file 1 [file Table_1.DOCX]

**Supplementary Table 1: Characteristics of patients with baseline LA enlargement in SCHypoT and euthyroid group before and after PS matching.**

| **Variables** | **Before PS matching** | | **SD** | ***P*-Value** | **After PS matching (1:2)** | | **SD** | ***P*-Value** |
| --- | --- | --- | --- | --- | --- | --- | --- | --- |
|  | **Euthyroid group (n=834)** | **SCHypoT group (n=207)** |  |  | **Euthyroid group (n=344)** | **SCHypoT group (n=185)** |  |  |
| **TSH (mIU/L)** | 2.19 (1.52-2.85) | 13.44 (10.66-16.40) |  | <0.001 | 2.34 (1.65-3.08) | 13.74 (11.24-16.40) |  | <0.001 |
| **Free T_3_ (pmol/L)** | 4.60 (4.20-5.00) | 4.20 (3.78-4.60) |  | <0.001 | 4.50 (4.00-4.90) | 4.20 (3.80-4.60) |  | 0.047 |
| **Free T_4_ (pmol/L)** | 17.20 (15.80-18.80) | 14.35 (12.63-16.63) |  | <0.001 | 17.05 (15.58-18.93) | 14.60 (12.70-16.50) |  | <0.001 |
| **Age (years)** | 57.6±11.5 | 59.5±12.0 | 0.162 | 0.041 | 58.6±10.4 | 59.0±12.3 | 0.034 | 0.714 |
| **Weight (Kg)** | 65.1±11.7 | 62.9±11.2 | -0.192 | 0.015 | 63.1±10.9 | 63.1±11.1 | -0.007 | 0.931 |
| **Height (cm)** | 164.7±8.6 | 162.7±8.5 | -0.229 | 0.003 | 163.5±8.8 | 163.0±8.4 | -0.053 | 0.569 |
| **Male (%)** | 475 (57.0) | 80 (38.6) | -0.375 | <0.001 | 143 (41.6) | 74 (40.0) | -0.033 | 0.726 |
| **Smoking (%)** | 149 (17.9) | 20 (9.7) | -0.239 | 0.004 | 34 (9.9) | 18 (9.7) | -0.007 | 0.955 |
| **Drinking (%)** | 97 (11.6) | 18 (8.7) | -0.096 | 0.228 | 26 (7.6) | 14 (7.6) | 0.000 | 0.997 |
| **Prior PCI (%)** | 10 (1.2) | 3 (1.4) | 0.018 | 0.730 | 5 (1.5) | 2 (1.1) | -0.035 | 1.000 |
| **Comorbidities** |  |  |  |  |  |  |  |  |
| **Hypertension (%)** | 306 (36.7) | 65 (31.4) | -0.112 | 0.155 | 108 (31.4) | 55 (29.7) | -0.037 | 0.692 |
| **Diabetes mellitus (%)** | 66 (7.9) | 23 (11.1) | 0.109 | 0.141 | 33 (9.6) | 17 (9.2) | -0.014 | 0.880 |
| **Congestive heart failure (%)** | 4 (0.5) | 1 (0.5) | 0.000 | 1.000 | 1 (0.3) | 0 (0.0) | -0.078 | 1.000 |
| **Coronary artery disease (%)** | 78 (9.4) | 28 (13.5) | 0.129 | 0.075 | 33 (9.6) | 21 (11.4) | 0.059 | 0.524 |
| **Atrial flutter/fibrillation (%)** | 288 (34.5) | 100 (48.3) | 0.283 | <0.001 | 141 (41.0) | 83 (44.9) | 0.079 | 0.390 |
| **Cerebrovascular disease (%)** | 50 (6.0) | 16 (7.7) | 0.067 | 0.359 | 23 (6.7) | 12 (6.5) | -0.008 | 0.930 |
| **Chronic kidney disease (%)** | 8 (1.0) | 6 (2.9) | 0.138 | 0.030 | 3 (0.9) | 1 (0.5) | -0.048 | 1.000 |
| **Chronic liver disease (%)** | 18 (2.2) | 8 (3.9) | 0.099 | 0.159 | 9 (2.6) | 5 (2.7) | 0.006 | 0.953 |
| **Medications** |  |  |  |  |  |  |  |  |
| **ACEI/ARBs use(%)** | 108 (12.9) | 36 (17.4) | 0.126 | 0.098 | 48 (14.0) | 25 (13.5) | -0.015 | 0.889 |
| **Statins use(%)** | 20 (2.4) | 7 (3.4) | 0.060 | 0.426 | 6 (1.7) | 5 (2.7) | 0.068 | 0.461 |
| **NYHA functional class** |  |  | 0.193 | 0.014 |  |  | 0.044 | 0.637 |
| **I/II (%)** | 352 (42.2) | 68 (32.9) |  |  | 128 (37.2) | 65 (35.1) |  |  |
| **III/IV (%)** | 482 (57.8) | 139 (67.1) |  |  | 216 (62.8) | 120 (64.9) |  |  |
| **Surgery Type** |  |  | 0.193 | 0.014 |  |  | 0.068 | 0.456 |
| **Single valve surgery (%)** | 450 (54.0) | 92 (44.4) |  |  | 166 (48.3) | 83 (44.9) |  |  |
| **Multiple valve surgery (%)** | 384 (46.0) | 115 (55.6) |  |  | 178 (51.7) | 102 (55.1) |  |  |
| **Follow-up period (months)** | 6.4±3.8 | 6.9±4.1 | 0.126 | 0.197 | 6.4±3.9 | 6.6±4.0 | 0.048 | 0.603 |

TSH, FT_3_, and FT_4_ data are expressed as median (IQR), while other data are expressed as mean ±standard deviation or n (%).

SCHypoT: subclinical hypothyroidism; PCI: percutaneous coronary intervention; ACEI: angiotensin-converting enzyme inhibitors; ARB: angiotensin receptor blockers; NYHA: New York Heart Association; LAD: left atrium PS: propensity score; SD: standardized differences.

**Supplementary Table 2: Characteristics of patients with baseline LA enlargement in OHypoT and euthyroid group before and after PS matching.**

| **Variables** | **Before PS matching** | | **SD** | ***P*-Value** | **After PS matching (1:4)** | | **SD** | ***P*-Value** |
| --- | --- | --- | --- | --- | --- | --- | --- | --- |
|  | **Euthyroid group (n=834)** | **OHypoT group (n=38)** |  |  | **Euthyroid group (n=86)** | **OHypoT group (n=31)** |  |  |
| **TSH (mIU/L)** | 2.19 (1.52-2.85) | 5.45 (4.68-6.45) |  | <0.001 | 2.20 (1.55-2.82) | 5.45 (4.66-6.46) |  | <0.001 |
| **Free T3 (pmol/L)** | 4.60 (4.20-5.00) | 4.40 (4.10-4.80) |  | 0.001 | 4.50 (4.20-4.90) | 4.50 (4.10-4.90) |  | 0.483 |
| **Free T4 (pmol/L)** | 17.20 (15.80-18.80) | 16.40 (14.80-18.40) |  | <0.001 | 16.85 (15.70-18.78) | 16.40 (15.10-18.35) |  | 0.015 |
| **Age (years)** | 57.6±11.5 | 59.5±10.0 | 0.179 | 0.310 | 59.9±11.4 | 60.5±9.2 | 0.058 | 0.591 |
| **Weight (Kg)** | 65.1±11.7 | 60.5±11.0 | -0.408 | 0.017 | 61.4±11.5 | 61.5±11.3 | 0.009 | 0.630 |
| **Height (cm)** | 164.7±8.6 | 161.2±8.4 | -0.403 | 0.019 | 161.1±8.5 | 162.1±8.8 | 0.116 | 0.920 |
| **Male (%)** | 475 (57.0) | 12 (31.6) | -0.529 | 0.002 | 36 (41.9) | 11 (35.5) | -0.132 | 0.535 |
| **Smoking (%)** | 149 (17.9) | 6 (15.8) | -0.056 | 0.743 | 15 (17.4) | 5 (16.1) | -0.035 | 0.868 |
| **Drinking (%)** | 97 (11.6) | 2 (5.3) | -0.228 | 0.301 | 8 (9.3) | 2 (6.5) | -0.104 | 1.000 |
| **Prior PCI (%)** | 10 (1.2) | 1 (2.6) | 0.103 | 0.389 | 3 (3.5) | 1 (3.2) | -0.017 | 1.000 |
| **Comorbidities** |  |  |  |  |  |  |  |  |
| **Hypertension (%)** | 306 (36.7) | 15 (39.5) | 0.058 | 0.728 | 36 (41.9) | 13 (41.9) | 0.000 | 0.994 |
| **Diabetes mellitus (%)** | 66 (7.9) | 2 (5.3) | -0.105 | 0.761 | 5 (5.8) | 2 (6.5) | 0.029 | 1.000 |
| **Congestive heart failure (%)** | 4 (0.5) | 0 (0.0) | -0.100 | 1.000 | 0 (0.0) | 0 (0.0) |  |  |
| **Coronary artery disease (%)** | 78 (9.4) | 3 (7.9) | -0.053 | 1.000 | 9 (10.5) | 3 (9.7) | -0.027 | 1.000 |
| **Atrial flutter/fibrillation (%)** | 288 (34.5) | 25 (65.8) | 0.659 | <0.001 | 47 (54.7) | 18 (58.1) | 0.069 | 0.743 |
| **Cerebrovascular disease (%)** | 50 (6.0) | 3 (7.9) | 0.075 | 0.498 | 6 (7.0) | 2 (6.5) | -0.020 | 1.000 |
| **Chronic kidney disease (%)** | 8 (1.0) | 1 (2.6) | 0.121 | 0.332 | 2 (2.3) | 1 (3.2) | 0.055 | 1.000 |
| **Chronic liver disease (%)** | 18 (2.2) | 2 (5.3) | 0.164 | 0.215 | 1 (1.2) | 2 (6.5) | 0.278 | 0.171 |
| **Medications** |  |  |  |  |  |  |  |  |
| **ACEI/ARBs use(%)** | 108 (12.9) | 7 (18.4) | 0.152 | 0.330 | 16 (18.6) | 6 (19.4) | 0.020 | 0.927 |
| **Statins use(%)** | 20 (2.4) | 0 (0.0) | -0.212 | 1.000 | 0 (0.0) | 0 (0.0) |  |  |
| **NYHA functional class** |  |  | 0.608 | 0.001 |  |  | -0.020 | 0.927 |
| **I/II (%)** | 352 (42.2) | 6 (15.8) |  |  | 16 (19.6) | 6 (19.4) |  |  |
| **III/IV (%)** | 482 (57.8) | 32 (84.2) |  |  | 70 (81.4) | 25 (80.6) |  |  |
| **Surgery Type** |  |  | 0.351 | 0.039 |  |  | 0.010 | 0.955 |
| **Single valve surgery (%)** | 450 (54.0) | 14 (36.8) |  |  | 31 (36.0) | 11 (35.5) |  |  |
| **Multiple valve surgery (%)** | 384 (46.0) | 24 (63.2) |  |  | 55 (64.0) | 20 (64.5) |  |  |
| **Follow-up period (months)** | 6.4±3.8 | 7.7±3.9 | 0.338 | 0.049 | 6.8±3.9 | 7.1±3.5 | 0.081 | 0.744 |

TSH, FT_3_, and FT_4_ data are expressed as median (IQR), while other data are expressed as mean ±standard deviation or n (%).

OHypoT: overt hypothyroidism; PCI: percutaneous coronary intervention; ACEI: angiotensin-converting enzyme inhibitors; ARB: angiotensin receptor blockers; NYHA: New York Heart Association; LAD: left atrium PS: propensity score; SD: standardized differences.
